# Three-Year Outcome of Full-Arch Fixed Prosthetic Rehabilitation through the All-on-4^®^ Concept Using Dynamic 3D Navigated Surgery (X-Guided™): A Retrospective Study

**DOI:** 10.3390/jcm13133638

**Published:** 2024-06-21

**Authors:** Armando Lopes, Miguel de Araújo Nobre, Inês Vitor

**Affiliations:** 1 Oral Surgery Department, Malo Clinic, Avenida dos Combatentes, 43, Level 9, 1600-042 Lisboa, Portugal; alopes@maloclinics.com; 2 Research, Development and Education Department, Malo Clinic, Avenida dos Combatentes, 43, Level 11, 1600-042 Lisboa, Portugal; ivitor@maloclinics.com

**Keywords:** edentulous jaw, dental implants, immediate loading, digital, all-on-4, navigated surgery

## Abstract

**Background/Objectives**: The insertion of dental implants using dynamic 3D navigated surgery while applying immediate function protocols for full-arch rehabilitations warrants further research. This study aimed to evaluate the outcomes of All-on-4^®^ rehabilitations using 3D Dynamic navigated surgery (X-Guide™). **Methods**: This study included 10 patients (women: 7; men: 3; average age: 59.9 years) rehabilitated with full-arch prostheses through the All-on-4^®^ concept, with 48 dental implants inserted using navigated surgery. The primary outcome evaluation was prosthetic/implant cumulative survival (CS), estimated using life tables. Secondary outcome evaluations were marginal bone resorption (MBR), biological complications, and mechanical complications. The evaluation parameters were measured between 1 and 3 years. **Results**: No patients were lost to follow-up. Two implants (4.2%) were lost in one patient (10%) with smoking habits, resulting in an implant CS rate of 95.8%. The average MBR was 0.51 mm ± 0.62 mm at the 1-year follow-up. The incidence rate of mechanical complications was 40% (*n* = 4 patients), all occurring in provisional prosthesis. No biological complications were registered. The patients maintained their prostheses in function throughout the follow-up of the study. **Conclusions**: Within the limitations of this study and based on the results, it can be concluded that the insertion of dental implants assisted by dynamic navigation for full-arch rehabilitation through the All-on-4^®^ concept may be a valid treatment alternative in the short-term follow-up. However, more studies are necessary to validate this treatment modality.

## 1. Introduction

Full-arch rehabilitation with fixed prosthesis supported by immediate-function protocols using tilted implants (All-on-4^®^, Nobel Biocare AB, Göteborg, Sweden) has demonstrated to be a viable treatment alternative with good short- and long-term outcomes [1,2]. Outcomes reported by recent systematic reviews and meta-analyses support the promising results registered in single-center retrospective studies, with the inclusion of tilted posterior implants together with axial implants in immediate function regarded as a safe procedure [2,3]. This conclusion can be drawn from the results, with high survival rates (>93.9%), low marginal bone loss (1.3 ± 0.4 mm from 12 to 60 months), and absence of significant differences between axial and tilted implants [2,3].

Considering the limitations of bone quality and quantity in atrophic edentulous jaws, the insertion of implants to secure a correct fixed prosthetic rehabilitation with immediate loading might be challenging. In the presence of these adversities, it is necessary to use systems that facilitate the surgical procedure aiming to increase the probability of success. Dynamic navigation consists of a combination of office-based imaging and complex simulation and planning software for predictable surgical procedures; in implant dentistry, it represents a tool providing real-time navigation, aiming for improved accuracy of implant placement, mainly through a passive optical motion tracking system with arrays [4]. This system has evolved from the previous generation of static-guided surgery, which, through CBCT imaging integration, allowed digital planning and production of surgical guides that enable the placement of dental implants using a flapless protocol, aiming for accuracy, customization, decreased pain or discomfort, and faster patient recovery [5,6]. This allowed modification of the surgical approach, from extensive flaps to allow visualizing the surgical area, to minimally invasive surgical interventions [5]. A further advantage consisted in planning the rehabilitations through a prosthetically driven approach, aiming for the best possible design of the prosthesis, better esthetics, optimized occlusion, and loading [5]. Nevertheless, these procedures are not complication-free, with the most common consisting of fractured surgical guides, amendments to the surgical plan, early implant failure due to lack of primary stability, or fractured prosthesis [5,6]. Furthermore, the early complication incidence rate could be as high as 9.1% and 18.8% for surgical and prosthodontic complications, respectively [7]. Concerning the clinical outcome, the survival of dental implants using guided surgery was reported in a range between 89% and 100% [8,9,10].

The use of more precise dynamic navigation systems can assist the surgeon by increasing the accuracy of implant placement relative to the planning, leading to more predictable surgical and prosthetic outcomes. Evaluating the existing literature [11,12], it is possible to assume that the use of dynamic navigation systems represents an added value in patients requiring rehabilitation of complete edentulism. This is possible given its high accuracy in implant positioning, providing a decrease in implant placement errors, with a 1% (95% CI: 0.00% to 2%) pooled prevalence of failures according to a systematic review and meta-analysis [12]. Considering the dynamic navigation systems for single-tooth implant-supported rehabilitations, a previous study reported an implant cumulative success rate of 98.1% and a marginal bone loss of 0.63 ± 0.25 mm after a 1-year follow-up [13]. However, in edentulous patients with the All-on-4^®^ treatment concept, the number of reports using dynamic navigation systems is scarce, limited to a case report [14]. The aim of this study was to evaluate the short-term clinical outcomes (prosthetic/implant survival, marginal bone resorption and complications) of implants placed with dynamic navigation assistance, through the All-on-4 concept, for support of fixed prostheses.

## 2. Materials and Methods

This retrospective study was performed in a private practice in Lisbon, Portugal and approved by an independent ethical committee (Ethical Committee for Health, Lisbon, Portugal; Authorization nº 002/2023, date of approval: 25 of September 2023). This study was carried out in agreement with the Declaration of Helsinki, and all patients provided written informed consent to participate. 

The study population consisted of patients in need of full-arch rehabilitations supported by implants in immediate function. The convenience sample of this study consisted of patients rehabilitated with full-arch prostheses through the All-on-4^®^ concept with implant insertion using 3D Dynamic navigated surgery, serving as the development group for the All-on-4 workflow. Inclusion and exclusion criteria were evaluated during treatment planning. The patients were included, provided they needed single full-arch fixed prosthetic rehabilitations through the standard All-on-4 concept (Nobel Biocare AB, Göteborg, Sweden) and were able to provide written informed consent for their participation. As exclusion criteria, patients with insufficient bone volume to perform a standard All-on-4, under active radiotherapy or chemotherapy treatment, or unable to provide written informed consent were excluded.

The surgery was performed following an implemented protocol [11,14]. The patients’ medical charts were evaluated, and clinical and radiographic supplementary exams were performed, including intraoral scan (Trios, 3Shape A/S, Copenhagen, Denmark), orthopantomography, and cone beam computerized tomography (CBCT) scan. The DTX Studio Implant Software V.3.6.4.2 (Nobel Biocare, Zurich, Switzerland) was used for baseline clinical evaluation and surgical/prosthetic planning, with the implant type and position selected based on the uploaded data (scanning and CBCT). The surgical procedure was performed by one surgeon with more than 16 years of experience (A.L.); prosthetic restoration was performed by the same team of experienced prosthodontists with 10 to 18 years of experience. The first implant was placed in January 2019 and the last in May 2022, and the patients were followed for 1 year. The surgical intervention was executed with the patient under local anesthetic effect (articaine chlorhydrate with 1:100,000 epinephrine; Scandinibsa^®^ 2%, Inibsa Laboratory, Barcelona, Spain) [14]. The medication protocol included the pre-surgical administration of diazepam (Valium^®^ 10 mg, Roche, Amadora, Portugal); corticosteroid medication (prednisone (Meticorten^®^ Schering-Plough Farma Lda, Agualva-Cacém, Portugal), 5 mg) administered daily in a regression mode (15 mg to 5 mg) between the day of surgery and 4 days post-operatively [14]; antibiotic therapy (amoxicillin 875 mg + clavulanic acid 125 mg, Labesfal, Campo de Besteiros, Portugal) administered 1 h before surgery and daily for 6 days thereafter [14]; anti-inflammatory medication (ibuprofen, 600 mg, Ratiopharm Lda, Carnaxide, Portugal) administered post-operatively between day 4 and day 7 [14]. Analgesics (clonixine (Clonix^®^, Janssen-Cilag Farmaceutica Lda, Barcarena, Portugal), 300 mg) were administered on the day of surgery and only in case of pain during the first 3 days post-operatively [14]. Antacid (Omeprazole, 20 mg, Lisbon, Portugal) was given between the day of surgery and post-operatively until day 6 [14].

**Figure 1:**
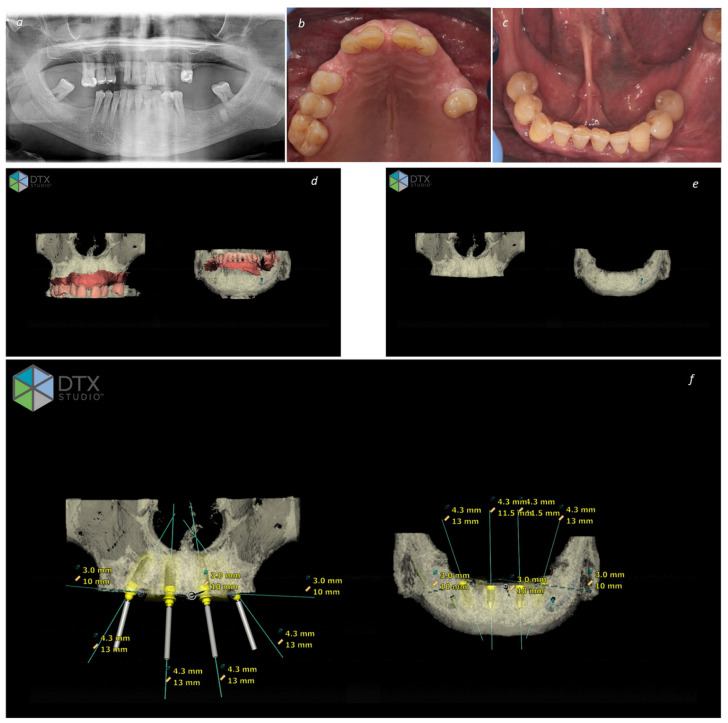
Planning a full-arch bimaxillary rehabilitation through the All-on-4 Concept: (**a**) Pre-treatment orthopantomography; (**b**) Pre-treatment intraoral photograph of the maxilla; (**c**) pre-treatment intraoral photograph of the mandible; (**d**) DTX Studio image exhibiting the pre-treatment condition in the maxilla and mandible with superimposition of CBCT and Intraoral Scanner (smart fusion); (**e**) DTX Studio image of the planning exhibiting the bone regularization to achieve a stable platform for implant insertion in the maxilla and mandible; (**f**) DTX Studio image of the final planning exhibiting the implant planning (diameter and length) in the maxilla and mandible.

**Figure 2:**
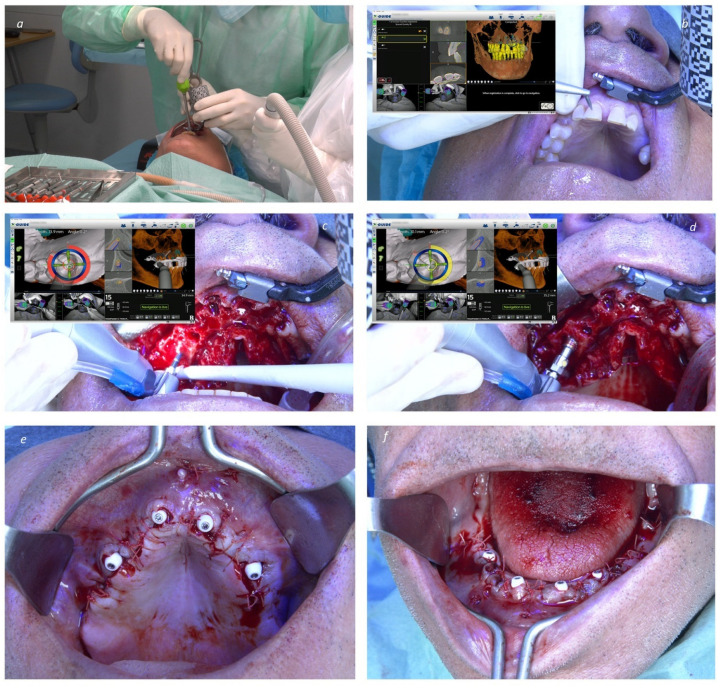
Peroperative bimaxillary full-arch rehabilitation through the All-on-4 Concept with implant insertion assisted by navigated surgery: (**a**) Connection of the X-Guide clip receptor and array; (**b**) Superimposition of the implant planning (performed in DTX Studio Implant Software) with the patient’s jaw was achieved through the selection of points in three teeth on the software (virtual) matched with a calibration probe on the real patient’s teeth at the same points (X-Mark); (**c**) 2 mm twist-drill during osteotomy for insertion of implant in position 15 (tilted implant); (**d**) Insertion of implant 15 (note the live feedback provided by the navigation in degrees and depth); (**e**) Intraoral photograph after implant insertion and abutment connection in the maxilla; (**f**) Intraoral photograph after implant insertion and abutment connection in the mandible.

**Figure 3:**
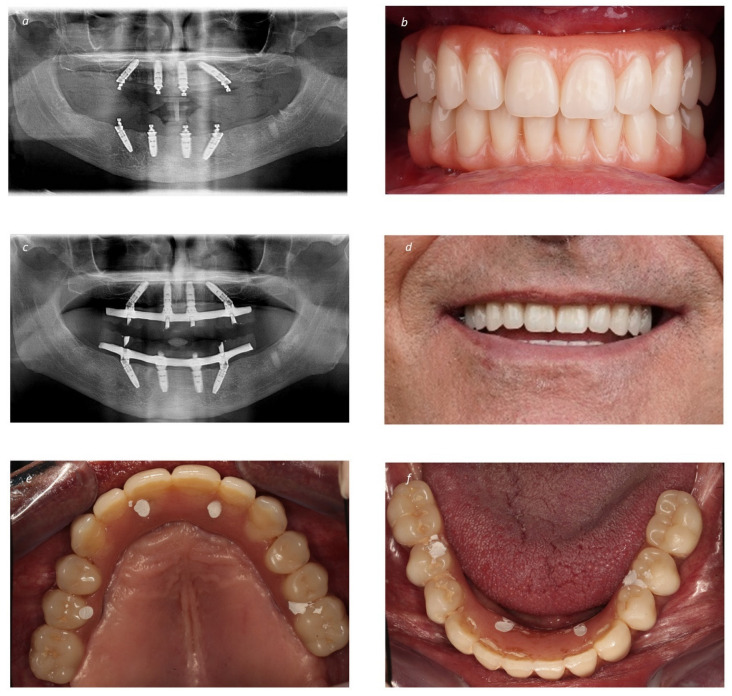
Prosthetic restoration of the bimaxillary full-arch rehabilitation: (**a**) Post-operative orthopantomography immediately after surgery; (**b**) Intraoral frontal photograph with the immediate provisional prostheses; (**c**) Final orthopantomography at 2 years of follow-up with the definitive prostheses (Maló Clinic Acrylic Bridge—titanium infrastructure, acrylic artificial gingiva and acrylic crowns); (**d**) Patient smiling with the definitive prostheses in place at 2 years of follow-up; (**e**) Intraoral occlusal view of the maxillary definitive prostheses at 2 years of follow-up; (**f**) Intraoral occlusal view of the mandibular definitive prostheses at 2 years of follow-up.

The X-Guide^®^ (Nobel Biocare AB, Göteborg, Sweden) enabled tracking of the surgical instruments relative to the patient in real time, with a computer display providing positional and guidance feedback. Before surgery, a tracker was attached to the patient’s jaw through 2 fixation screws and a holder. Another tracker was attached to the contra-angle. The X-Guide system only works when both trackers are simultaneously under both cameras. Superimposition of the implant planning with the patient’s jaw (performed in DTX Studio Implant Software), was achieved through the selection of points on three teeth in the software (virtual), matched with a calibration probe in the patient’s teeth (clinically) at the same points (X-Mark). If the patient was fully edentulous, 3 to 5 fiducial screws were screwed into the bone before the CBCT scan, and before surgery started, the same calibration probe allowed the matching of these screws in the software and in the mouth. After the successful superimposition process, a mucoperiosteal flap was raised along the top of the ridge with buccal relieving incisions in the molar area (maxillary cases). Unviable teeth and roots were extracted, followed by curettage for extraction socket cleaning. The crest was regularized with the objective of creating a stable and leveled platform. The precision drill, the 2.0 mm drill, the 2.4–2.8 mm drill, and the 3.2–3.6 mm drill were calibrated before the osteotomy, considering the spatial position provided by the live navigation. The two posterior implants were inserted next to the anterior wall of the sinus (maxillary rehabilitations), or anterior to the mental foramina (mandibular rehabilitations), in both cases using 30–45 degrees of distal tilting; while the 2 anterior implants were inserted in an axial position in both maxillary and mandibular rehabilitations. The posterior implants emerged bilaterally at the second premolar/first molar positions; the anterior implants emerged bilaterally at the lateral incisor position. All implants achieved an insertion torque greater than 35 N/cm before setting. Multi-unit abutments were connected to the implants (posterior tilted implants: MultiUnit 30 degrees; anterior axial implants: MultiUnit straight abutments; Nobel Biocare AB, Göteborg, Sweden). All osteotomies and implant placements were dynamically guided through the X-Guide^®^ software (8012) 3.4.2.3 (Nobel Biocare, Göteborg, Sweden). Flap closure was performed using non-resorbable sutures (3-0, B Braun Silkam, Aesculap Inc., Center Valley, PA, USA). Immediate function was achieved by connecting a full-arch pre-made immediate provisional prosthesis on the same day as the surgery. The prostheses were made of high-density acrylic resin (PalaXpress, Kulzer, Hanau, Germany) with 10 teeth (Mondial and Premium teeth, Kulzer, Hanau, Germany). The patients were instructed in their diet (soft food diet) and hygienic self-care for 4 months, using a post-operative toothbrush (Elgydium Clinic 7/100, followed by 15/100 after 2 weeks, Pierre Fabre Dermo Cosmetique, Lisboa, Portugal) and a chlorhexidine gel (Elugel, Pierre Fabre Dermo Cosmetique, Lisboa, Portugal). Clinical evaluation together with prophylactic measures were performed at day 10 post-operatively, at 2 months, 4 months and 6 months (corresponding to the functional osseointegration period), and thereafter every 6 months. A clinical situation is presented in Figure 1, Figure 2 and Figure 3.

The primary outcome measures considered in the present study were prosthetic/implant survival. Prosthesis success was based on function: if a prosthesis remained in function without the need to be replaced, it was considered a success. Dental implant success was determined by the following criteria: clinical stability (the prostheses were removed at each evaluation appointment: 10 days, 2 months, 4 months and 6 months, 1 year, 2 years and 3 years); fulfilled purported function without any discomfort to the patient; absence of infection or suppuration; and absence of radiolucent areas around the implants. The secondary outcomes evaluated were marginal bone resorption and the incidence of mechanical and biological complications. Marginal bone resorption was evaluated using the patient and the implants as the unit of analysis through periapical radiographs taken at implant insertion and 1 year post-operatively. An outcome assessor examined all implant radiographs, assessing the marginal bone level through an image analysis software (ImageJ version 1.40 g for Windows, National Institutes of Health, Bethesda, MD, USA). A conventional radiograph holder was used and manually adjusted to achieve an estimated orthognathic position of the film. Reading was performed using the implant platform as the reference point (the horizontal interface between the implant and the abutment). Marginal bone resorption was defined as the difference in marginal bone level at 1 year, relative to the bone level at the time of surgery. The acceptance/rejection of the radiographs was based on the clarity of the implant threads, assuming that a clear thread guarantees both an orthogonal direction and sharpness of the radiographic beam towards the implant axis. 

The authors assessed the incidence of mechanical complications (fracture or loosening of mechanical and prosthetic components) and biological complications (soft tissue inflammation, fistula or abscess formation).

The statistical analysis included the estimation of cumulative implant survival (life tables) and descriptive statistics for the marginal bone resorption and incidence of complications. The data were analyzed using the software SPSS for Windows version 26 (IBM SPSS, New York, NY, USA).

## 3. Results

### 3.1. Sample Selection

This study included 10 patients (three men and seven women), with an age range of 37 to 80 years (mean 59.9 years), serving as a development group. A total of eight patients had conditions according to the International Classification of Disease, version 11 (ICD-11): Three patients were smokers (30%), five patients (50%) presented a cardiovascular condition, one patient (10%) presented a thyroid condition, two patients (20%) presented high cholesterol levels, two patients (20%) presented depression, one patient (10%) presented Crohn’s disease, and one patient (10%) was treated for opioid addiction; four patients (40%) presented more than one condition.

### 3.2. Prostheses and Implants

A total of 12 full-arch prostheses were connected: six maxillary rehabilitations, two mandibular rehabilitations, and two bimaxillary rehabilitations. A total of 48 implants were inserted (*n* = 6 Nobel Speedy Groovy, *n* = 24 Nobel Parallel CC, *n* = 18 Nobel Parallel CC TiUltra; Nobel Biocare AB, Gothenburg, Sweden), with the following distribution according to the jaw: 32 implants inserted in the maxilla and 16 implants inserted in the mandible.

### 3.3. Implant Survival Rate

**Figure 4:**
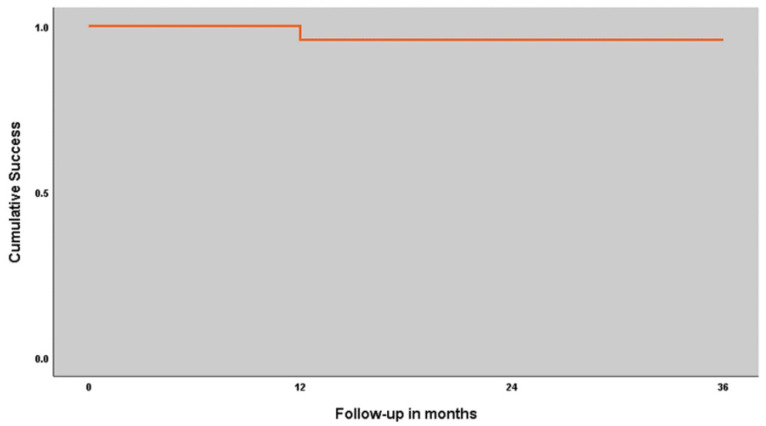
Cumulative survival rate for the implant-supported fixed prostheses: A 95.8% cumulative success rate was registered during the follow-up of the study (up to 3 years).

No patients were lost to follow-up. Two implants were lost in one female patient with aged 55 years with smoking habits: both implants presented mobility (implant in position 15 after 7 months, and implant in position 25 after 9 months of follow-up). Both implants were removed, resulting in an implant cumulative survival rate of 95.8% with a follow-up of up to 3 years (Table 1; Figure 4). The failed implants were replaced by a standard implant (position 15) on the same day as implant failure and a zygomatic implant (position 25) four months after the failure. The prosthesis remained in function supported on the remaining implants throughout the replacement of the failed implants (the prosthesis was sectioned at position 25 when the implant was lost). The replacement implants were not accounted for in this study.

### 3.4. Marginal Bone Loss

**Figure 5:**
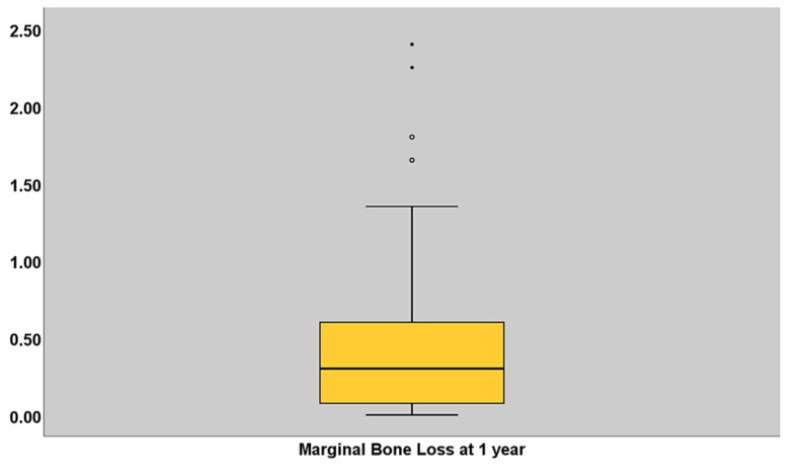
Boxplot illustrating the marginal bone loss measured in millimeters at 1 year of follow-up. The median is represented by the horizontal black line inside the box; the lower box edge represents the 25th percentile; the upper box edge represents the 75th percentile; whiskers represent the standard deviation; dots represent outlier values.

Eighty-five percent of the implant radiographs were readable for marginal bone loss at 1 year. The average marginal bone loss was 0.51 mm ± 0.62 mm at the 1-year follow-up (Table 2; Figure 5).

### 3.5. Mechanical Complications

Mechanical complications were registered in 4 patients (40%) during the provisional prosthetic phase: Three patients (30%) fractured the provisional prostheses, and 1 patient (10%) presented abutment screw loosening. The occlusion was adjusted for all patients, the abutment screw was retightened, and the provisional prostheses were mended in the dental laboratory. No further mechanical complications occurred. No biological complications were registered.

## 4. Discussion

The present study is the first performed with full-arch navigated surgery and also has the longest follow-up (up to 3 years) in a treatment modality with sparse studies. Our study registered a cumulative implant survival rate of 95.8% with up to 3 years of follow-up for implants placed in immediate function using a dynamic navigation system. 

The survival rate registered in the present study is comparable to other publications using freehand and static computer-assisted techniques. Concerning freehand surgical procedures, a systematic review and meta-analysis evaluating the outcomes of maxillary and mandibular rehabilitations through the All-on-4^®^ treatment concept reported cumulative implant survival rates of 97.6% and 98.7% at 3 years for the maxilla and mandible, respectively [2]. Concerning previous-generation guided surgery procedures, a systematic review and meta-analysis evaluating static computer assistance in healed sites with 18 publications and 2675 implants reported implant survival rates between 89% and 100% at 1-year [15]. Judging by the results, the present study is within the expected range for the All-on-4 concept (Nobel Biocare AB, Göteborg, Sweden) and indicates that this rehabilitation alternative may be viable in the short term. 

Two implants failed in one patient during the osseointegration phase. The patient in question presented smoking habits, a fact that might potentially explain the outcome: Smoking has a deleterious effect on the survival of dental implants. Two previous systematic reviews evaluating the effect of smoking habits on the outcomes of dental implant treatments, using a pool of 107 studies and 80,300 implants [16] and using a pool of 292 publications and 150,108 implants, registered failure rates in smokers up to 140% higher when compared to non-smokers [17]. 

The marginal bone loss registered in this study compares favorably to other publications on the All-on-4 concept at the same evaluation time point. In a clinical study on the All-on-4 concept using computer-guided flapless implant surgery, a mean marginal bone resorption of 1.9 mm at the 1-year follow-up was reported [18]. Considering the non-guided approach, the increased marginal bone resorption reported in older studies, ranging between 0.6 mm [1] and 0.9 mm [19], denotes a probable improvement in the protocols associated with this rehabilitation procedure, including the implants’ micro-design (surface) and the effect of the learning curve. When compared to a recent protocol using exclusively implants with an ultra-hydrophilic anodized surface that registered 0.39 mm of marginal bone loss at 1 year [20], the result of the present study is comparable. 

The overall 40% mechanical complication rate finds parallel with a systematic review and meta-analysis [10], reporting fracture of provisional and definitive prostheses and screw loosening as the most frequent prosthetic complications. Other complications included extensive occlusal adjustments, abutment and prosthetic misfit, and midline deviation [10]. In the present study, the high percentage of mechanical complications may derive from all patients presenting implant-supported fixed prostheses as opposing dentitions, a factor previously related to the increase of mechanical complications [21]. Considering this result, it is deemed necessary for patients with these characteristics to have a shorter recall regimen, with regular control of the occlusion. Nevertheless, all situations were resolved, and the patients maintained their prostheses in function throughout the study follow-up. 

In this study, despite the 30% prevalence of smokers, no biological complications were registered. Despite the benefits of the recall regimen applied in the present study, the result should be interpreted with caution as it might only be the reflection of a short-term follow-up. Peri-implant disease is set to be a time-dependent factor, with an increased probability of incidence as the follow-up increases [22]. 

Considering that both navigational implant placement and conventional freehand implant placement offer similar survival rates, peri-implant outcomes, and prosthetic functionality, the potential benefit of using dynamic navigated implant surgery resides in the possible elimination of challenges and complications associated with freehand implant placement, including, for example, damage to the inferior alveolar nerve, floor of mouth hematoma, damage to adjacent roots, sinus infections secondary to inadvertent sinus perforations, fractured implants due to off-axis loading, poor esthetics secondary to thin buccal bone, or soft tissue interproximal bone loss secondary to placing implants too close to adjacent teeth and implants [4].

The limitations of this study include being conducted in a single center, a reduced sample of 10 patients (representing a development group), absence of sample size calculation, the short follow-up time, and the absence of a control group; therefore, the interpretation of the results should be made with caution. Nevertheless, no loss to follow-up occurred, contributing to the robustness of the internal validity of the study. Future research should focus on the evaluation of soft tissue clinical outcomes, using a larger sample, a control group without assisted navigation, and longer follow-up. Furthermore, the assessment of implant placement accuracy and its implications for implant and prosthetic survival, marginal bone resorption, and surgical/prosthetic complications is an important parameter that will allow a comprehensive evaluation of the dynamic navigation protocol.

## 5. Conclusions

Considering the results, the insertion of dental implants assisted by dynamic navigation for full-arch rehabilitation through the All-on-4 Concept may be a valid short-term treatment option. More studies with larger sample sizes, longer follow-ups, and more robust designs are necessary to validate this treatment modality for full-arch fixed prosthetic rehabilitation.

## Figures and Tables

**Table 1:** Life table estimating the cumulative implant survival rate.

Time	Implants				Survival Rate (%)	Cumulative Survival Rate (%)
	Total	Failed	Lost to Follow-Up	Not Yet Due		
Placement—1 year	48	2	0	0	95.8%	95.8%
1–2 years	46	0	0	30	100%	95.8%
2–3 years	16	0	0	8	100%	95.8%

**Table 2:** Marginal bone resorption at 1 year of follow-up.

**Average (mm)**	0.51	
**Standard Deviation (mm)**	0.62	
**Number**	40	
**Frequencies**	***n***	**%**
**0–1.0 mm**	34	85%
**1.1–2.0 mm**	4	10%
**2.1–3.0 mm**	2	5%

## Data Availability

Data access will be provided by the authors upon reasonable request.
